# The Charging Events in Contact-Separation Electrification

**DOI:** 10.1038/s41598-018-20413-1

**Published:** 2018-02-06

**Authors:** Umar G. Musa, S. Doruk Cezan, Bilge Baytekin, H. Tarik Baytekin

**Affiliations:** 1 UNAM-National Nanotechnology Research Center, Bilkent University, 06800 Ankara, Turkey; 2 Chemistry Department, Bilkent University, 06800 Ankara, Turkey

## Abstract

Contact electrification (CE)—charging of surfaces that are contacted and separated, is a common phenomenon, however it is not completely understood yet. Recent studies using surface imaging techniques and chemical analysis revealed a ‘*spatial*’ bipolar distribution of charges at the nano dimension, which made a paradigm shift in the field. However, such analyses can only provide information about the charges that remained on the surface after the separation, providing limited information about the actual course of the CE event. Tapping common polymers and metal surfaces to each other and detecting the electrical potential produced on these surfaces ‘*in-situ*’ in individual events of *contact* and *separation*, we show that, charges are generated and transferred between the surfaces in both events; the measured potential is bipolar in *contact* and unipolar in *separation*. We show, the ‘contact-charges’ on the surfaces are indeed the net charges that results after the *separation* process, and a large contribution to tribocharge harvesting comes, in fact, from the electrostatic induction resulting from the generated CE charges. Our results refine the mechanism of CE providing information for rethinking the conventional ranking of materials’ charging abilities, charge harvesting, and charge prevention.

## Introduction

Contact electrification (CE) is the electrical charge development on two identical^1,2^ or different^3,4^ materials during their contact and separation. Charge build-up on surfaces results in ‘zaps’ or electrical discharges, which are detrimental for some industries (e.g. electronics, space and aviation, plastic manufacturing) causing millions of dollars losses annually. On the other hand, electrophotography^5^, electrostatic coating, filtration, separations^6^, and triboelectric energy harvesting (generators)^7,8^ benefit from charges developed during CE. Although CE is so common, our fundamental understanding on its mechanism is still incomplete, even millennia after its discovery. CE depends on contacting material’s physical and chemical properties, environmental conditions and the nature of the physical contact between materials, and therefore ambiguous results are encountered in the related literature, hampering the uncovering of its mechanism^9,10^. The event gets even more complicated when mass transfer (exchange of bits of charged material between contacting surfaces) is involved^11,12^. Recent discoveries made by using chemical and surface analysis techniques, however, provided a large step-forward in contact electrification research, for instance; (1) charging of two identical surfaces questioned the idea of pure electron transfer mechanism and undermined the concept of building up of a general *triboelectric series*^13–16^ (2) the observation of nano-to-macro oppositely-charged charge domains on surfaces^17–25^ disproved the previously assumed homogenous charge distribution on surfaces after contact, (3) the measurement of charges in nano domains showed that the electric potential on surfaces is ca. 100 times more than previously thought^17^. Based on these new findings, it was proposed that the charge generation is closely related to mechanically generated chemical species; radicals, cations, and anions formed after bond-breakages on surfaces^17,26–31^. Also, three fundamental transfer processes, electron^32,33^, ion^16,34,35^, and material transfer^11,12^ are now reconsidered in terms of bond-breaking upon plastic deformations^31,36–38^. Some groups further investigated the chemical events on tribocharged surfaces such as surface-modifications^39,40^, different types of bond-breaking processes^26–30^, and other chemical changes like surface oxidation, showing their role in contact electrification^37^. Here we show, the above-mentioned ‘spatial resolution’ of charging event (imaging of surface charge at meso to nano^17–25^ scales, and tracking of material transfer and chemical changes), can now be augmented from the results obtained by the ‘temporal resolution’ of the event, the monitoring of charge formation, transfer, and dissipation in the separate events of *contact* and *separation*, to obtain a more universal mechanism of contact electrification.

## Results and Discussion

### Contact-separation cycles of polymer-metal electrification, and simultaneous potential measurement

We used a tapping device set at different tapping frequencies (1–10 Hz), which allows the contact and separation of two surfaces (a polymer and a metal), for individual detection of the electrical potentials produced during contact and separation of polymer/metal surfaces. In our experiments, we used various polymers; polydimethylsiloxane (PDMS), polytetrafluoroethylene (PTFE), polysulfone (PSU), polyvinyl chloride (PVC), polypropylene (PP), kapton, polyacetate, polyethylene terephthalate (PET), polycarbonate (PC), and Nylon. Metals used were aluminum (Al), copper (Cu) and stainless steel. Of the two contacting surfaces, the first one is the surface of the metal stub (ME) directly attached to a 100 mega ohm (input impedance) oscilloscope probe, and the second is a polymer that is mounted on another metal stub (BE), which is attached to an identical probe. A two-channel oscilloscope was used to independently measure the open circuit electrical potential (in volts) generated during the contact and separation of the two surfaces. Current measurements were performed using a low-noise current preamplifier (SR570 Current Preamplifier, Stanford Research Systems, Inc.) (For details about the experimental parameters, see SI).

### Resolution of the *contact* and *separation* signals

**Figure 1:**
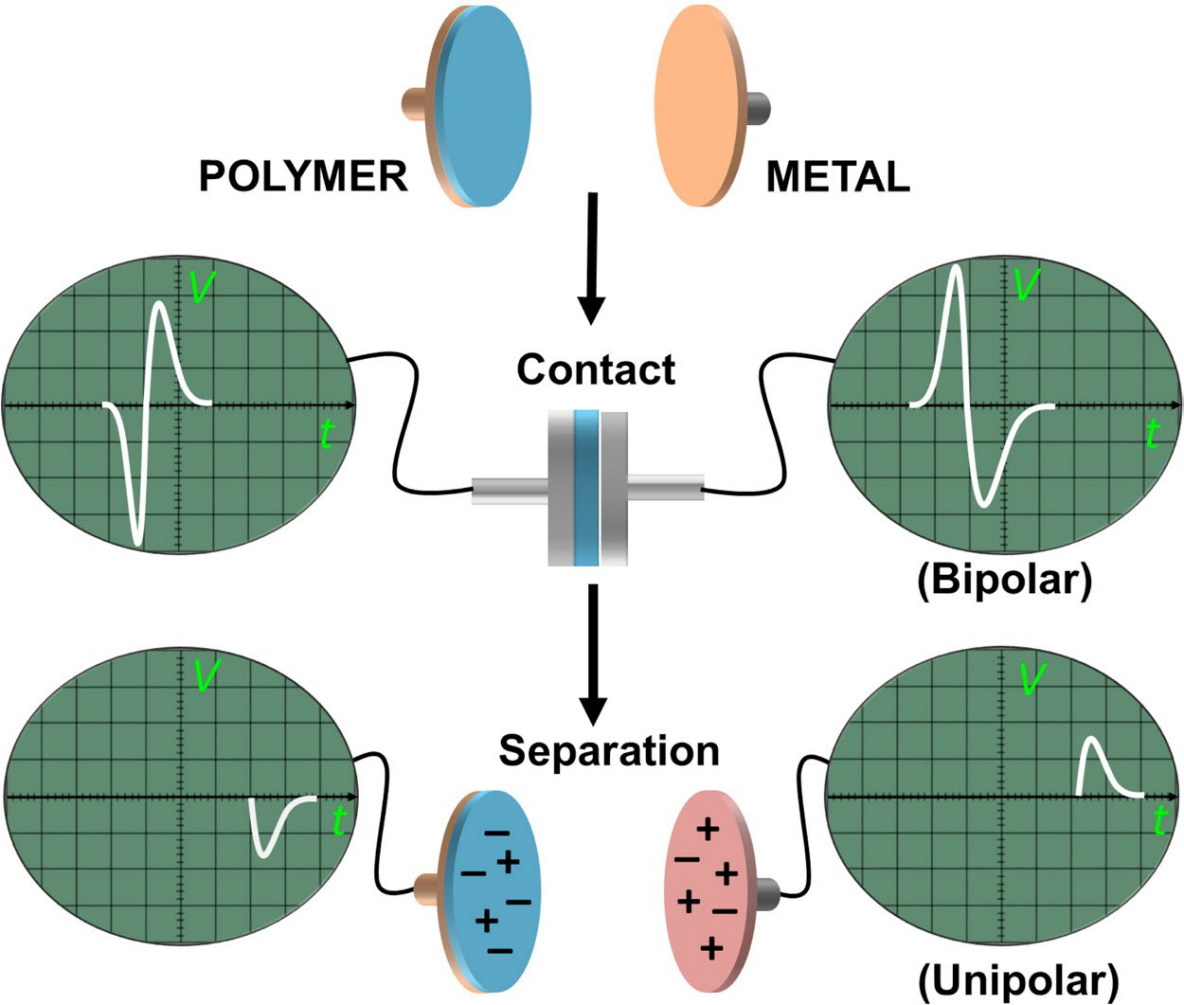
Schematic representation of polymer-metal contact/separation electrification and the corresponding oscilloscope signals (electrical potential) for the individual events of contact and separation. Electrical potential signals are bipolar (+ and −) at contact and unipolar at separation. In the experimental setup, contacting surfaces are; a metal base electrode (***BE***) covered with a thick (100 μm) polymer film and a metal electrode (***ME***, Al, Cu, or stainless steel). The charges created during contact and separation measured as electrical potential on the metal electrodes were collected using 100 mega ohm oscilloscope probes connected to a two-channel oscilloscope (OWON SDS7072, 70 MHz, 2 + 1 Channel, 1 GS/s). The channels of the oscilloscope can measure electrical signals independently. The distance between the electrodes was adjusted by an x-y stage and separation force was provided by a spring. A microprocessor (Arduino nano) was used to set the frequency of tapping and the force was applied by a solenoid actuator. See SI for further details of the setup and Movie S1 for the simultaneous visual observation of the contact and separation, and their corresponding electrical signals.

**Figure 2:**
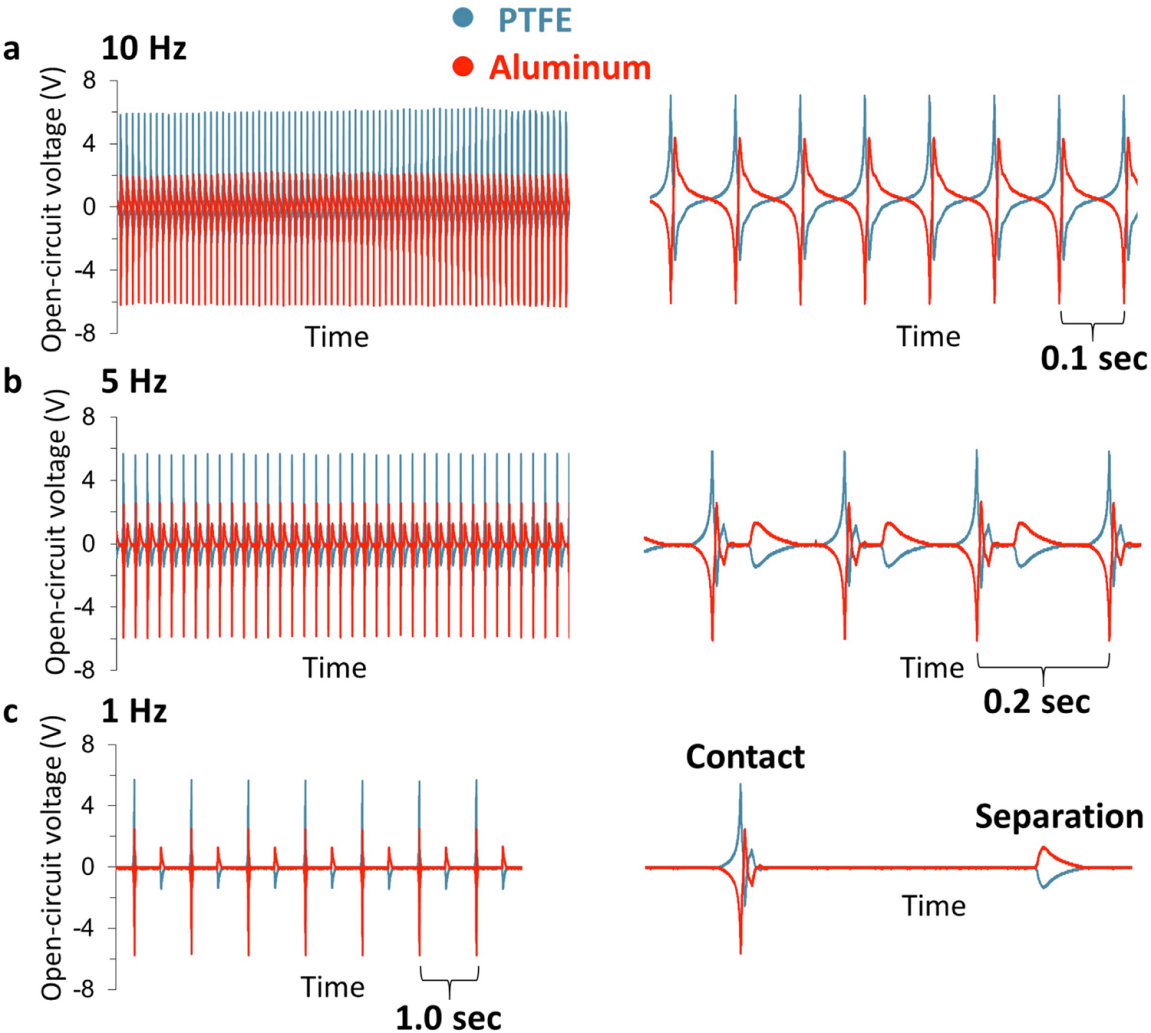
Resolving the signals generated at *contact* and *separation* during a typical contact-separation cycles of a polymer and a metal. Polymer-metal (PTFE on ***BE***, Aluminum as ***ME***) contact electrification at (**a**) 10 Hz, (**b**) 5 Hz, and (**c**) 1 Hz tapping frequencies, showing a drastic dependence of signal pattern of open-circuit voltages on tapping frequency. (The signals, ‘as obtained’ on the left column, and ‘zoomed in’ on the right column). At 10 Hz, the signals for the two electrodes, ***BE*** and ***ME***, appear as two peaks; which can be attributed ‘purely’ to contact and separation processes in each cycle, respectively. However, as the tapping slows down to 5 Hz and later to 1 Hz, and the contact and separation events are ‘resolved’, bipolar signals for contact, and unipolar signals for separation emerge. RH = 13.5%, T = 23 °C.

**Figure 3:**
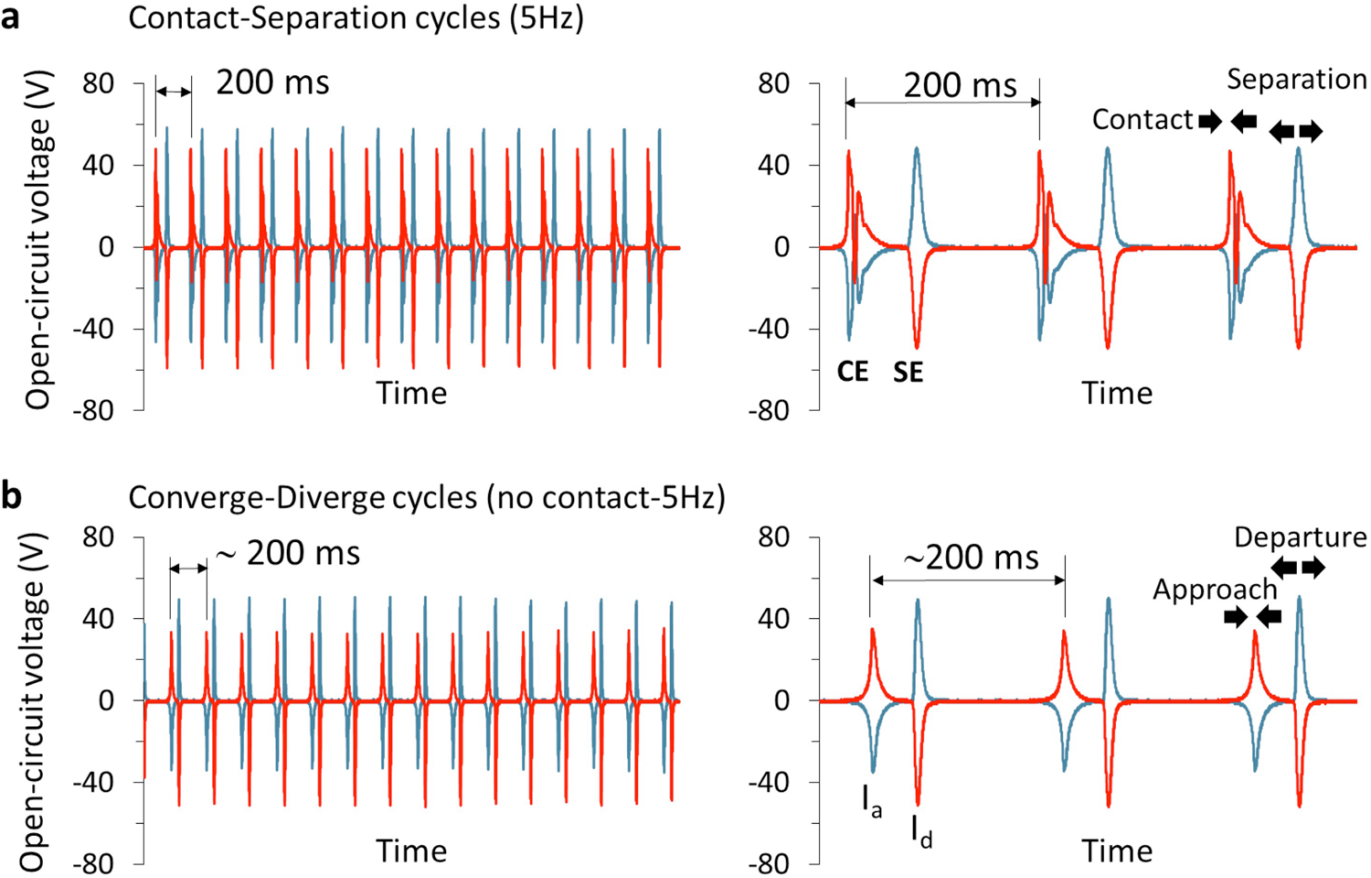
(**a**) Identifying the signals for *contact electrification*. Open circuit potential signals that were generated due to CSE and electrostatic induction during contact mode, (**b**) Open circuit potential signals that were generated due to electrostatic induction during non-contact mode. Aluminum metal and polymer (PSU) were used in the experiment and frequency of CSE tapping in (**a**) and oscillation in (**b**) were set to 5 Hz (RH = 19%). (The signals, ‘as obtained’ on the left column, and ‘zoomed in’ on the right column).

**Figure 4:**
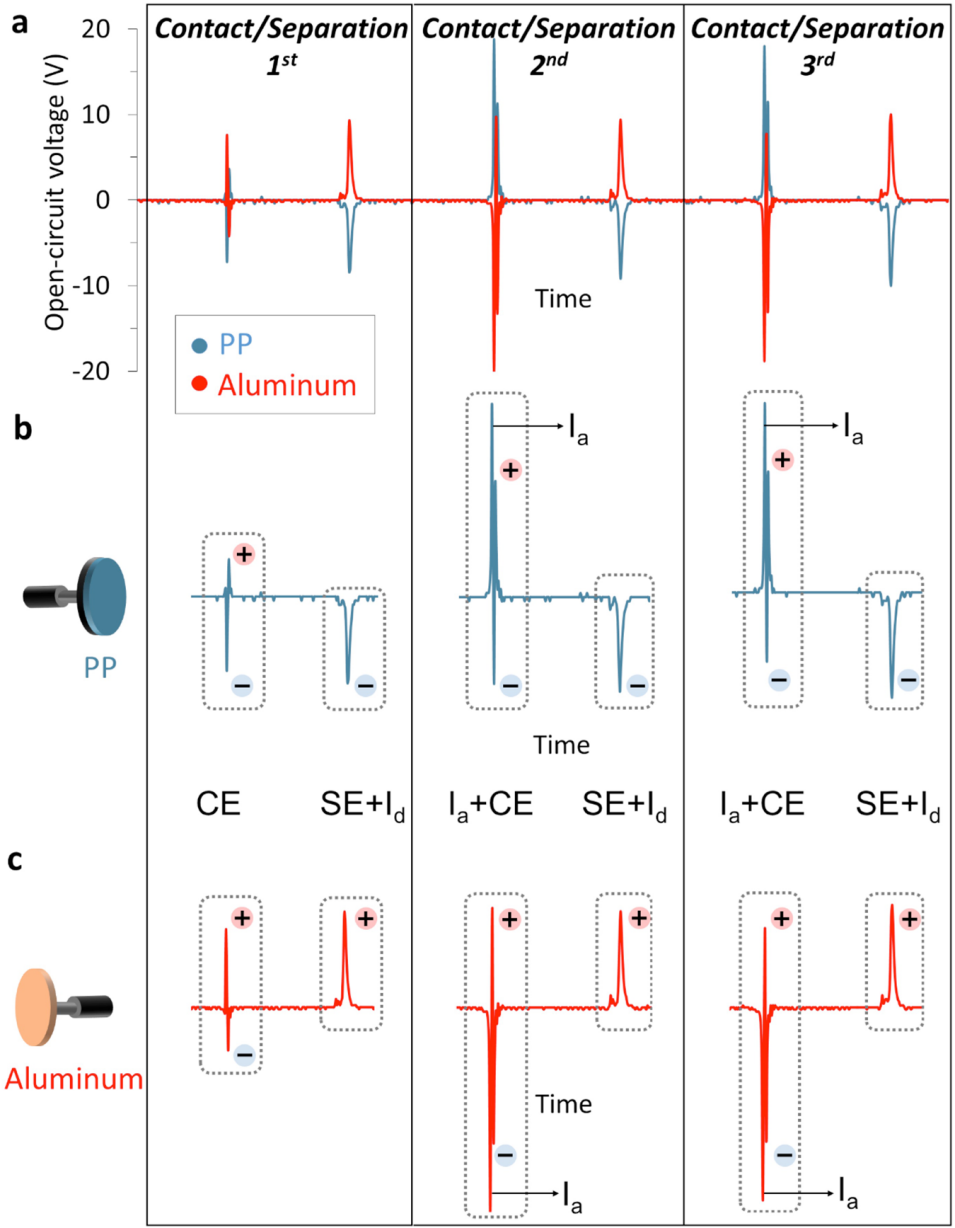
Analysis of electrical potential signals of consecutive contact/separation cycles upon metal-polymer contact. (Here, data shown for PP on *BE*-Aluminum as *ME*, at 1 Hz). (**a**) Overall signals generated at the first three contact/separation cycles. (**b**) Signals for PP (*BE*), and (**c**) signals for Al (*ME*). Individual events of electrostatic induction upon approach (I_a_), contact electrification (CE), separation (SE), and electrostatic induction upon departure (I_d_) are labelled. As expected, I_a_ signal is missing at the first contact, and I_d_ appears after first SE (since the surfaces becomes charged already after SE).

As illustrated in Fig. 1, we first tapped polytetrafluoroethylene, PTFE (100 micron thickness, mounted on base electrode, ***BE***) and aluminium (as metal electrode, ***ME***), at three different tapping frequencies (10 Hz, 5 Hz, and 1 Hz), simultaneously monitoring the events and their respective signal outputs on the oscilloscope screen by a video camera (Movie S1, Fig. 1). For all other polymer/metal pairs see Fig. S1. In all cases (Fig. 2 and Fig. S1), the two output channels show almost equal but opposite charging of the materials on the two electrodes, evidenced by the comparison of the measured electrical potential, current and the charge measured after separation (Figs S1–S3). The results show, however, as the contact/separation frequency is reduced from 10 Hz to 1 Hz, the shapes of the two output signals change dramatically, i.e., more than two signals for both electrodes emerge: Contact event creates bipolar charging (with alternating + and − voltages), whereas separation leads to a unipolar charging (either + or −). Here, we note that the bipolar signal during contact is not due to the mechanical vibrations or slipping during contact, and is a true electrical signal as evident from Movie S2. We also note that the signal patterns are not affected significantly by the thickness of the polymer sample (15–100 μm for various polymers) or by the change in relative humidity (10–35%). The effect on any material transfer on the obtained signals upon polymer-metal tapping is discussed in SI, Figs S11 and S12. We have also performed tapping experiments with various metal-polymer combinations at the BE electrode (tapping all BE’s at 5 Hz to Al (ME)), to account for any differences in signals that may emerge from the differences in the interface resistance of various metal polymer contact at the BE. The basic form of open-circuit potential signals generated upon contact/separation cycles does not change upon changing the type of metal on BE, (Fig. S8a)—in all possible combinations, induction, followed by the bipolar charging upon contact, and subsequent unipolar separation signal (see below) can be detected. The BE metals’ resistance is found to play an insignificant role also in the rate of discharge upon contact charging event (Fig. S8b). Reducing the frequency lower than 1 Hz did not change the signal patterns significantly, so we believe that already at tapping frequency of 1 Hz, the events are well-resolved in terms of their signal patterns. (Since the data for the polymer/metal pairs are very similar, throughout the rest of the text (Figs 2–4) we show data from different polymer/metal pairs).

To verify that the appearance of bipolar signal is due to charging alone and not the transport imbalances of the electrons, we have also tapped to *two identical BE electrodes*, namely PVC-PVC, PC-PC, PTFE-PTFE, PP-PP, PDMS-PDMS, and kapton-kapton (each polymer was mounted on Al on BE) (Fig. S9). Similar electrical signal patterns were observed in these cases with identical material contact, too, only with a smaller values of contact/separation charges (detected as open-circuit electrical potentials) as expected from identical material contacts^1,17^.

Although a variety of signal patterns for different tapping frequencies, consecutive positive and negative peaks in voltage and current, is very common in the related literature^41–47^, since the focus of most studies is on energy harvesting, or other applications, most studies do not concentrate on the assignments of *all* the obtained electrical signals. If the signals are obtained at tapping frequencies ≥10 Hz (as in Fig. 2a), their interpretations might be made as the two peaks of voltage for each electrode, per cycle—first one (+ or − only) for *contact*, and the second one (− or + only, reverse polarity of the contact signal) for separation. This is because of the overlap of the signals from individual charging events, as shown in Fig. 2. Another reason might be the effect of the large electrostatic induction signal compared to the signals that originate only from contact electrification. ‘Bipolar’ contact and ‘unipolar’ separation signals, which are only possible to get with higher resolution, were also reported previously, but they were not described in detail, since the motivation of these studies were usually to increase the power output^45^. To the best of our knowledge, this is the first study to understand the ‘bipolar’ contact and ‘unipolar’ separation signals, linking them to actual events of contact/separation, and electrostatic induction.

### Identifying the signals that originate from individual *contact*, *separation* and *induction* events

In order to understand the origins of signals, and the interesting bipolar pattern (alternating + and − voltages) of electrical potential signal during contact/separation electrification that emerges as the frequency of tapping is reduced during contact/separation (Fig. 2, Movie S1), we did the following experiments. In the first experiment (Fig. 3), we allowed the pristine polymer and metal surfaces to contact and separate at 5 Hz (Fig. 3a). As the contact/separation cycles were being performed, we suddenly adjusted the distance between polymer and metal surfaces to 500 microns by manipulating the x-y stage, so that the polymer and the metal electrode could not physically contact each other anymore in the subsequent approach/departure cycles (Fig. 3b). Careful comparison of signal output patterns in Fig. 3a,b show that the signals for approach/departure cycles (no contact, Fig. 3b), look similar to those obtained from the contact/separation cycles (Fig. 3a), but they lack the signals for *contact* at each cycle. (Again, this difference can only become clear as the signals are diligently inspected) From this difference, we conclude that the first and the largest peaks in the ‘contact’ signals in contact/separation cycles (Figs 2c, 3a) are due to electrostatic induction of the charge by the surfaces charged in previous cycles. (Induction effect also exists in separation signals in contact/separation cycles, as discussed in SI text and Figs S4 and S5). This implies, the signals obtained during conventional contact/separation cycles are not solely due to the contact/separation event, and that induction plays a major role (see Fig. S5 for its relative contribution), as also mentioned in the previous literature. More importantly, this experiment shows us that the consecutive, oppositely charged signals (‘bipolar’ signals) only appear when there is contact of surfaces during the cycles—apparently, these are the signals for *contact electrification*.

In a second experiment for investigation of electrical signals that originate from the contact/separation electrification, we let this time polypropylene (PP) and aluminium (Al), initially uncharged, go through contact/separation cycles at 1 Hz (Fig. 4). In this experiment, the signal pattern remains almost identical for hundreds of cycles after the second contact; therefore, we show the representative signals from the first three contact/separation cycles (Fig. 4a). The interpretation of the signals obtained upon contact/separation of the polymer and the metal can be summarized as follows: Since we start with uncharged surfaces, an induction-free first contact gives rise to two consecutives oppositely charged signals (bipolar charging), which we will continue to refer as electrification during contact or *contact electrification* (CE) (Fig. 4). In the second and subsequent contacts, CE signals are accompanied by the large signal of electrostatic induction (I_a_) (as verified in Fig. 3), since the surfaces are already charged from the previous cycles. Separation gives rise to a separate unipolar ((+) or (−)) electrical signal, as also shown in Fig. 4, which we will refer to as electrification during separation, or *separation electrification* (SE). SE signals contain the electrostatic induction signals (as a result of surface charges after SE) for departure (I_d_). Similar signal patterns can be obtained for other pairs; polymer-metal (Fig. S1), with reversal of polarity for Nylon (6,6) or polycarbonate (PC)/metal pairs (Fig. S2), and also for polymer/polymer pairs (Fig. S6).

### Bipolar contact signals through bond-breaking events

Appearance of the bipolar contact and unipolar separation signals are quite surprising according to the conventional theories on mechanism of contact/separation electrification, which assume a ‘unidirectional charge transfer’ between surfaces when they contact. The ‘unidirectional charge transfer’ idea has so far been especially pronounced for metal-polymer contacts, in which the electron transfer is proposed to occur from metal to polymer, for the polymers that gain (−) charge. On the principle of unidirectional charge transfer, common materials had been ranked according to their ‘tendencies of electron transfer’, forming ‘triboelectric series’, that still serves as a basic understanding of CE. Recently, using surface imaging techniques Kelvin Probe, KPFM, EFM, force microscopy, and chemical analysis, we and others^17–25^ have shown that positively and negatively charged domains (bipolar charge distributions) ranging from nano to macro exist on common dielectric surfaces after CE. Although these studies cannot provide information about the direction of charge transfer during the event, they indirectly show that there could be a bidirectional charge transfer during the charging events. Moreover, they also pointed out that the charge formation is related to the bond-breaking events that create mechanoions. These species can also play a role in the charge transfer.

**Figure 5:**
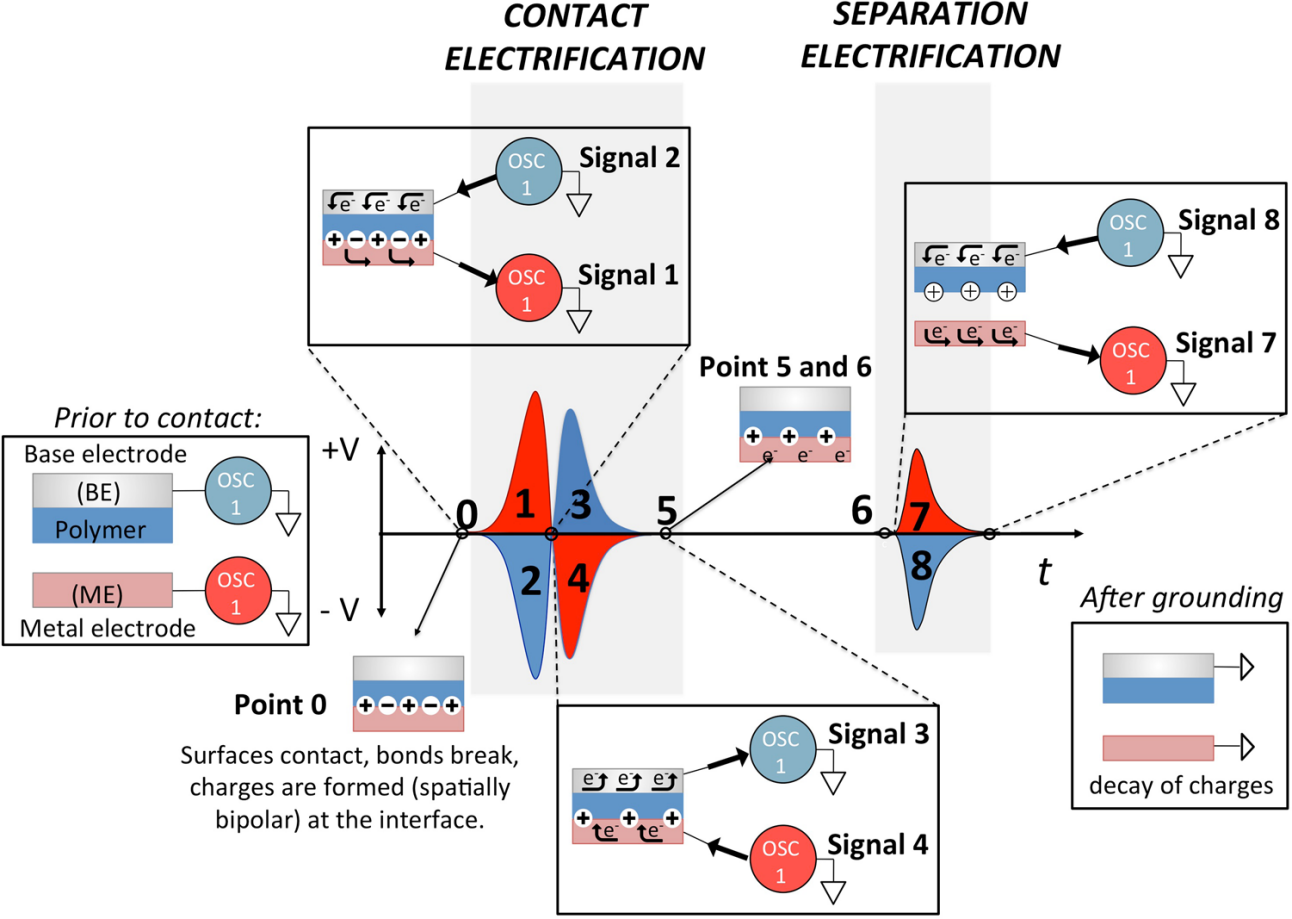
Proposed mechanism of contact electrification based on metal-polymer interaction. Typical electric potential signal patterns of contact and separation event in CE of a polymer and a metal surface (Tapping frequency 1 Hz, shown here signals obtained from CE of PP and Al, large induction signal during approach is omitted for clarity). Corresponding events for signals formed during contact (signals 1–4) and separation (signals 7 and 8). The ‘backflow’ of electrons causing signals 3 and 4 cannot take place during separation, since the surfaces are physically separated. CE charges decay if the electrodes are grounded.

We should note again that appearance of signals during contact and separation events is a definite indication of charge transfer between surfaces during these events. Although it is impossible to fully define the roles of species (electrons, mechanoions etc.) involved in such a transfer, standing on the recent evidences on charge formation by bond-breaking and material transfer, one might guess about the molecular level events. For example, in our case, the observation of bipolar charging (bidirectional transfer) at contact implies that the transfer mechanism should be more complicated than the previously thought unidirectional electron transfer (e.g. as in Movie S3) and that recently proposed mechanism of charge formation through bond-breaking might help in proposing a hypothetical mechanism. Using this recent knowledge, and following the course of signal appearances in typical signal patterns of polymer/metal contacts that generate negatively charged polymer surfaces at separation, a schematic for the transfer of charges in contact/separation electrification can be surmised as shown in Fig. 5. For simplifying the explanation, we exclude the signals due to electrostatic induction (I_a_ and I_d_) and keep only the signals due to contact/separation processes (CE and SE) (Fig. 5). Before contact, both surfaces are in the uncharged state (Fig. 5a, point 0). During the first contact, charges are created bipolarly (conserving the total charge as, X^+^ = Y^−^ + e^−^, X and Y mechanoions, e^−^ free electrons) at the polymer/metal interface because of the bond-breaking processes on the polymer’s surface as was previously verified in the literature^17,18,26,31,36^. 36 and also in Fig. S10, Movie S4. At this moment, the generated free electrons (e^−^) flow from the surface of the ***BE*** to Osc1 (channel 1) (Fig. 5, signal 2), giving rise to a simultaneous electron flow from Osc2 to the metal (***ME***) (Fig. 5, signal 1). From the comparison of intensities of signal 1 and 2 (Fig. S5), we realise that the polymer/metal interface is net positive at the *contact*. Since the polymer surface at the interface is still net positive, it initiates a ‘back flow’ of electrons from Osc 1 to ***BE*** (Fig. 5, signal 3) which produces a concurrent electron flow form metal to Osc2 (signal 4). Now, there is a charge balance at the surface and there is no more net flow (Fig. 5, point 6). If the initial charges formed on the polymer surface were not bipolar, the mechanism would result in only one unipolar ‘contact charge’ signal (either positive or negative) and not to a bipolar temporal voltage signal. Here we stress again that the **temporal** bipolar contact charging can only be observed, if there is such a **spatial** bipolar charging. During separation, positive and negative charges are generated again as a result of further bond-breakages (as also supported by a detectable amount of material transfer that occurs between the surfaces)^11^. These initiate the subsequent electron flow from/to the electrodes, which are detected as signals 7 and 8. But now, a ‘back flow’ of electrons cannot happen, since the surfaces are physically separated. Therefore, negative charges remain at the polymer’s surface (for polymers that end up with net negative surface charge, Fig. S1), while positive charges remain on the surface of ***ME***. We note that for the polymer surface, neutralization happens through charge decay in air (in hours, when isolated), charges on the metal surface decay rapidly, only when/if the system is connected to ground (Fig. 5). In continuous tapping, such charge decay is interrupted by the subsequent cycle, and starting from the second cycle, each cycle starts with charged surfaces, which induce opposite charges at each electrode and hence the I_a_ signals are generated (Fig. 4). So far we described the case of polymer/metal contacts that generate *negatively* charged polymer surfaces at separation. In the typical signal patterns of polymer/metal contacts that generate *positively* charged polymer surfaces at separation (Fig. S2), the I_a_, CE and SE, I_d_ signal polarities are reversed w.r.t. the above case; upon contact, the interface is net negative for these contacts, as can be seen from the comparison of the intensities of corresponding signals 1 and 2.

To summarize, we have separated and assigned, previously unresolved signals of contact and separation electrification individually by slowing down the contact/separation cycles. The analysis of the signal patterns verified that the largest peak in the pattern is due to induction of charges that happens during approach of the previously charged surfaces. The first time analysis of the generated electrical signals in individual events of contact and separation showed that signals generated at contact are bipolar (both positive and negative) and separation signals are unipolar (positive or negative). Although the signal polarity depends on the nature of the polymer, the contact and separation are bipolar and unipolar, respectively, for all metal/polymer and polymer/polymer contacts showing a universal behaviour. Our results might be used to extend the knowledge on the newly emerging chemistry-based mechanism that is built on observation of bipolar surface charges on dielectric materials at the nano and macro scales, which so far could give information on charges only after separation. As pointed out earlier^48^, explorations of variations in charge transfer during the contact/separation event provide useful hints for finding the general mechanism of charge formation/transfer. Therefore, we believe that the electrical signals that are obtained *in situ* can provide information about the ‘true’ mechanism of contact/separation electrification, which is essential especially for building up revised triboelectric series as well as understanding and developing technologies such as charge harvesting by energy converters, sensor applications, or making new generation antistatic materials based on actual mechanism of contact electrification.

## Electronic supplementary material

Supplementary Information

Movie S4

Movie S1

Movie S2

Movie S3
