# Supplementary material for: The Charging Events in Contact-Separation Electrification
Source: Sci Rep. 2018 Feb 6;8:2472. doi: 10.1038/s41598-018-20413-1 (PMC5802787; doi:10.1038/s41598-018-20413-1)
Supplement: Supplementary file 1 — Supplementary Information [file 41598_2018_20413_MOESM1_ESM.pdf]

SUPPLEMENTARY INFORMATION

The Charging Events in Contact-Separation Electrification

Umar Gishiwa Musa, S. Doruk Cezan, Bilge Baytekin, and H. Tarik Baytekin**

U. Gishiwa Musa, Asst. Prof. Bilge Baytekin, Asst. Prof. H. Tarik Baytekin
UNAM-National Nanotechnology Research Center, Institute of Materials Science and Nanotechnology, Bilkent University, 06800 Ankara, Turkey
E-mail: baytekin@unam.bilkent.edu.tr

Doruk Cezan, Asst. Prof. Bilge Baytekin
Bilkent University, Department of Chemistry
06800 Ankara, Turkey

Keywords: contact electrification • tribocharging • polymer electrification • triboelectric generators • static electricity

EXPERIMENTAL DETAILS

Polymer films and their preparations: Polymers that were used in this study are polydimethylsiloxane (PDMS), polytetrafluoroethylene (PTFE), polysulfone (PSU), polyvinyl chloride (PVC), polypropylene (PP), kapton, polyacetate, polyethylene terephthalate (PET), polycarbonate (PC), and Nylon. PDMS (Dow, Sylgard 184) films were produced by thorough mixing of elastomer and curing agent at 10:1 weight ratio using a mixer and waiting for 2 hours under low pressure before curing. For obtaining the desired thickness of polymers, we varied the amounts of prepolymer mixture that was poured into polystyrene Petri dishes. These mixtures were then heated in an oven under vacuum at 65 ^{0}C for 10 hours. After curing, PDMS films are cut and removed from the petri dishes and the samples were smoothly laid on the surfaces of Al SEM stubs of three different diameters (6, 12, and 18 mm, respectively) for the experiments. All the other types of polymers were purchased from McMaster. Thickness values of the polymers samples were between 15-100 micron. Al and Cu were used as based electrode (***BE***) and metal electrode (***ME***) in the experiments.

Voltage and Current Measurements: In order to investigate contact electrification at any given frequency, we developed a reliable and virtually low noise mechanical tapping device. This device was controlled by a home-made system using a microcontroller (Arduino Nano) and some additional electronic components (2N3904 NPN transistor, 1N4007 diode, and resistors etc.). The device was operated in contact and separation modes horizontally, such that one electrode was held fixed and the other electrode periodically contacted the fixed one.

We measured open circuit voltage and open circuit current (Fig S3) of the contact and separation electrification (CSE) using our tapping device at 10 Hz, 5 Hz, and 1 Hz frequencies, respectively. All potential data were taken directly using a digital oscilloscope (Owon SDS7072 70 MHz, 100 mega ohm impedance, 2+1 Channel, 1 GS/s**)** with P4100 Series probes (100:1/100 MHz, Input voltage 2KV, P4100 series high voltage probes). Current measurements were performed using a low-noise current preamplifier (SR570 Current Preamplifier, Stanford Research Systems, Inc.) monitored by using SDS7072 oscilloscope and digitalized using its software. Charge measurements were performed using Keithley 6517 electrometer that is connected to a homemade Faraday cup. A 4189 Traceable Humidity/Thermometer–Control was used to measure the relative humidity and the temperature throughout the study. (RH=10-35%, T=20-25 ^{o}C).

Origin of the observed potential signals. The static charges accumulated on surfaces upon metal-polymer or polymer-polymer contacts have been studied in two ways: 1) Immersing the contact-charge surfaces in a Faraday cup attached to an electrometer that can directly display charge (Q), 2) By measuring the electrical potential signals using the high-internal resistance (in our case 100 MΩ) probes of an oscilloscope, which are connected to electrodes on which polymer samples are mounted. The high internal resistance of the probes let no current flow through the measurement device (oscilloscope), thus the potential measured is called the 'open-circuit potential'. (($V_{OC} = (\Delta\sigma_{SC}.S)/C$), where S is the area of one electode, $\Delta\sigma_{SC}$ the transferred charge density, C is the capacitance between the two electrodes S. Wang, Y. Xie, S. Niu, L. Lin, Z. L. Wang, *Adv. Mater.* **2014**, *26*, 2818–2824). This is to make sure that the measured potential is the one created at the (contact or separation) electrification of the surfaces. Explicitly, the origin of the signals is the electron (charge) flow from contact- or separation-charged surfaces to the electrodes or vice versa, which causes a difference in the open-circuit potential (which is then displayed as a signal on the oscilloscope), since V_{OC} is proportional to the charge density. This second mode is very common to retrieve electrical potential signals from such tapping and has been used in innumerous devices called triboelectric generators, explicitly discussed in the book Wang Z. L., Lin L., Chen J., Niu S., Zi Y. *Triboelectric Nanogenerators*, Springer (2016).

Induction Signal Observed at Separation: In the main text, Fig 4, we have shown the contribution of charge induction arising from the charges (created at the separation of the previous cycle) on the signals observed for *contact*. Similarly, charges formed at separation contribute also to the signals observed at the separation: There are two interfering signals (one appears as a separate signal or a shoulder on the broader signal) for the separation (Fig S4, Fig 3 in main text). We surmise that the first signal for separation is due to electrification (the actual charging event upon separation) and the broad one is due to electrostatic induction (I_d) at separation and during divergence of the electrodes. We have shown that even the first separation generates sufficient surface charges for electrostatic induction, and thus, unlike the 'first contact', which is free of the induction signal, first separation electrification

causes a detectable induction signal for this separation event. Also, the overall signal observed at the separation event appears broader than those at the contact for every cycle. The reason for this difference might be the different timescales of contact and separation electrifications due to mechanical reasons, e.g. asymmetric push-pull behavior of actuator, or pronounced adhesion forces as in the case of PDMS-metal pair.

SUPPLEMENTARY FIGURES

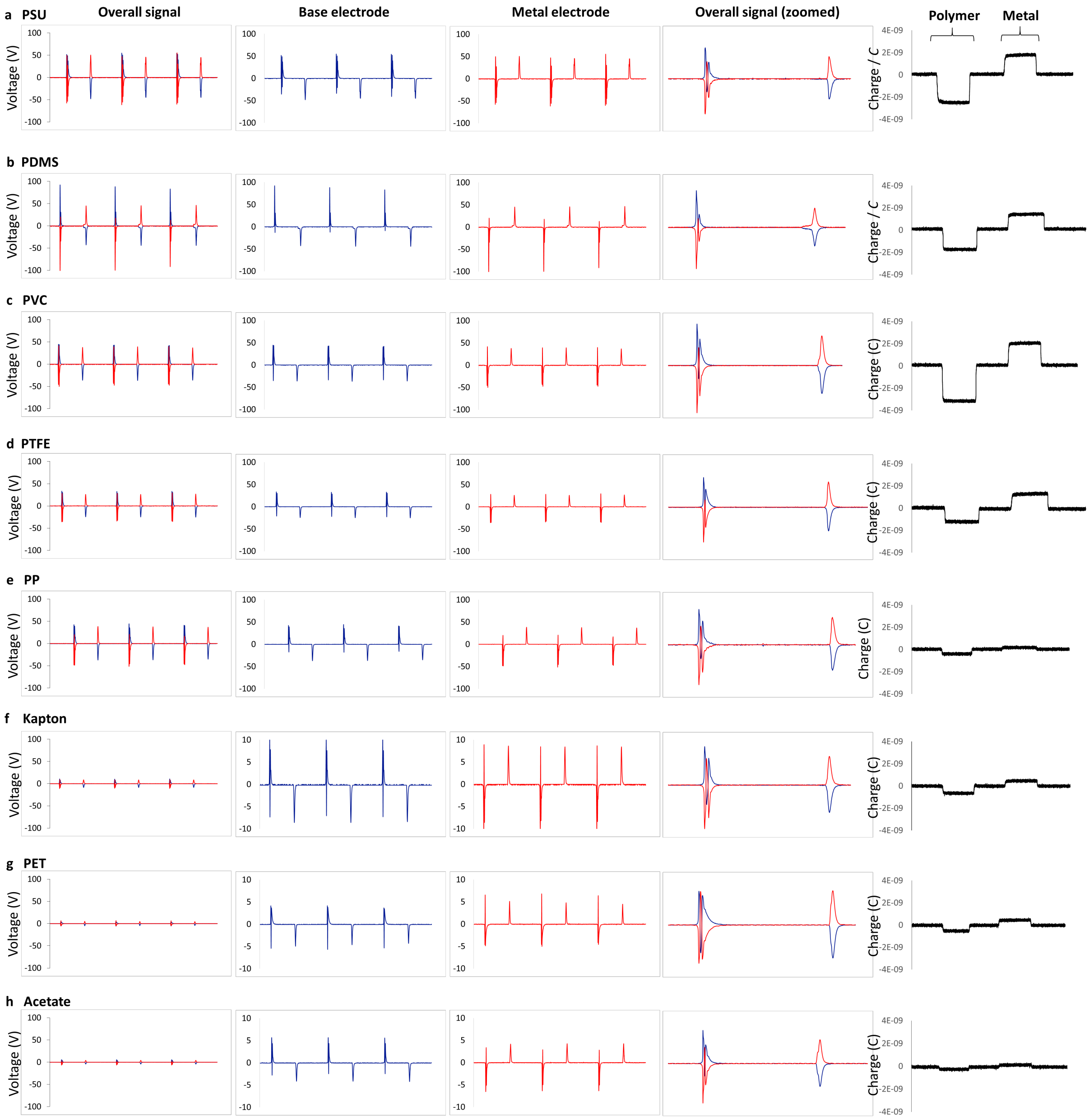

Fig S1 Open circuit contact potential and triboelectric charging signals (far right column) that are generated due to contact and separation electrification (CSE) and electrostatic induction (I_a and I_d) between aluminum metal and different polymers **a** PSU, **b** PDMS, **c** PVC, **d** PTFE, **e** PP, **f** Kapton, **g** PET, and **h** polyacetate, tapping at 1 Hz. CSE and I_a and I_d potential signals were obtained using oscilloscope and tapping device. Negative triboelectric charge generated on polymers and positive triboelectric charges on metal electrodes were measured separately, immersing the surfaces into a cylindrical Faraday cup respectively after the separation (far right column), using Keithley 6517 electrometer.

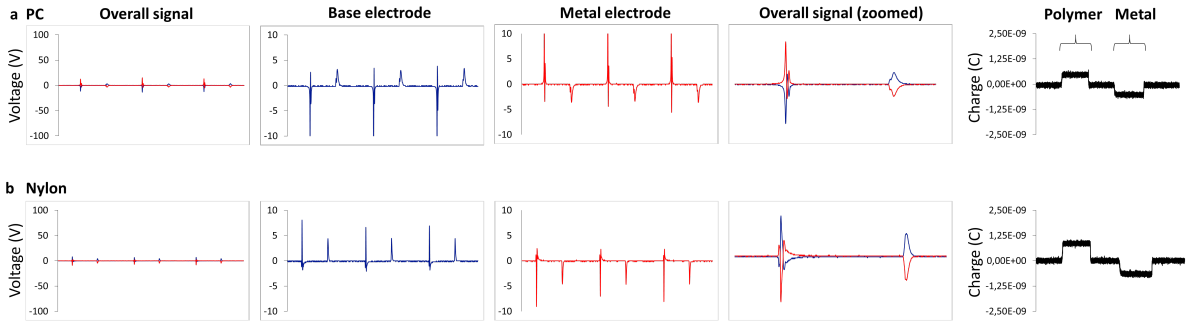

Fig S2 Open circuit contact potential and triboelectric charging signals (far right column) that are generated due to contact and separation electrification (CSE) and electrostatic induction (I_a and I_d) between aluminum metal and different polymers (a) Nylon and (b) PC, tapping at 1 Hz. CSE and I_a and I_d potential signals were obtained using oscilloscope and tapping device. Positive triboelectric charge generated on polymers and negative triboelectric charges on metal electrodes were measured separately, immersing the surfaces into a cylindrical Faraday cup respectively after the separation (far right column), using Keithley 6517 electrometer.

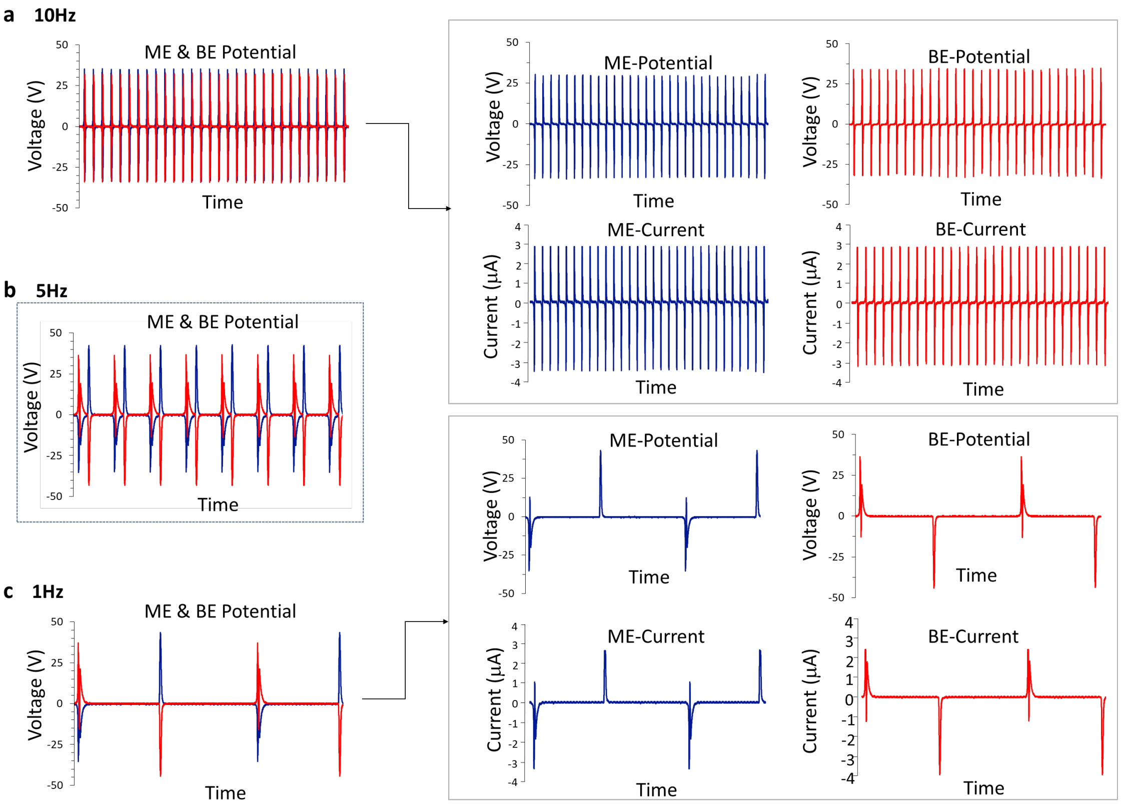

Fig S3 Open circuit **current** and open circuit **potential** signals that were generated during contact and separation electrification (CSE) and electrostatic induction (I_a and I_d) for the tapping between aluminum metal and polymer (PSU) at 10 Hz, 5 Hz, and 1 Hz. (RH=19%).

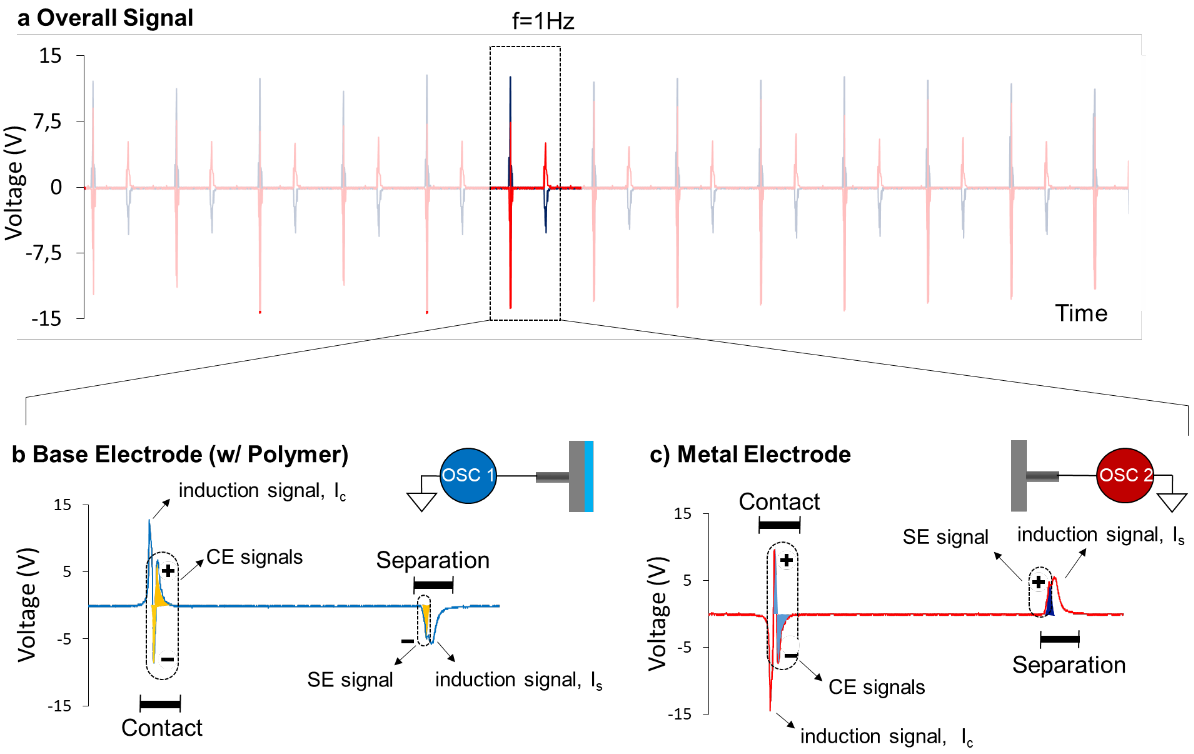

Fig S4 Assignment of individual events (contact electrification, separation electrification and induction) causing the observed electrical signals at a contact/separation cycle. **a** Generated overall potential signal for subsequent cycles at 1 Hz. Enlarged view of the signals at **b** ***BE*** (base electrode attached to polymer) and **c** ***ME*** (metal electrode) during contact and separation events showing a symmetric output w.r.t each other. In both patterns contact signals (CE signals) are bipolar (alternating + and -), associated with an initial signal from induction caused by the approach of surfaces charged in the previous cycle (I_a). Separation signals (SE signals) are unipolar and they, too, include a contribution from induction upon departure (I_d) (Here shown; PDMS on ***BE***-Aluminum as ***ME***, electrification signals purely from contact and separation electrification are marked with yellow in (b) and and blue in (c))

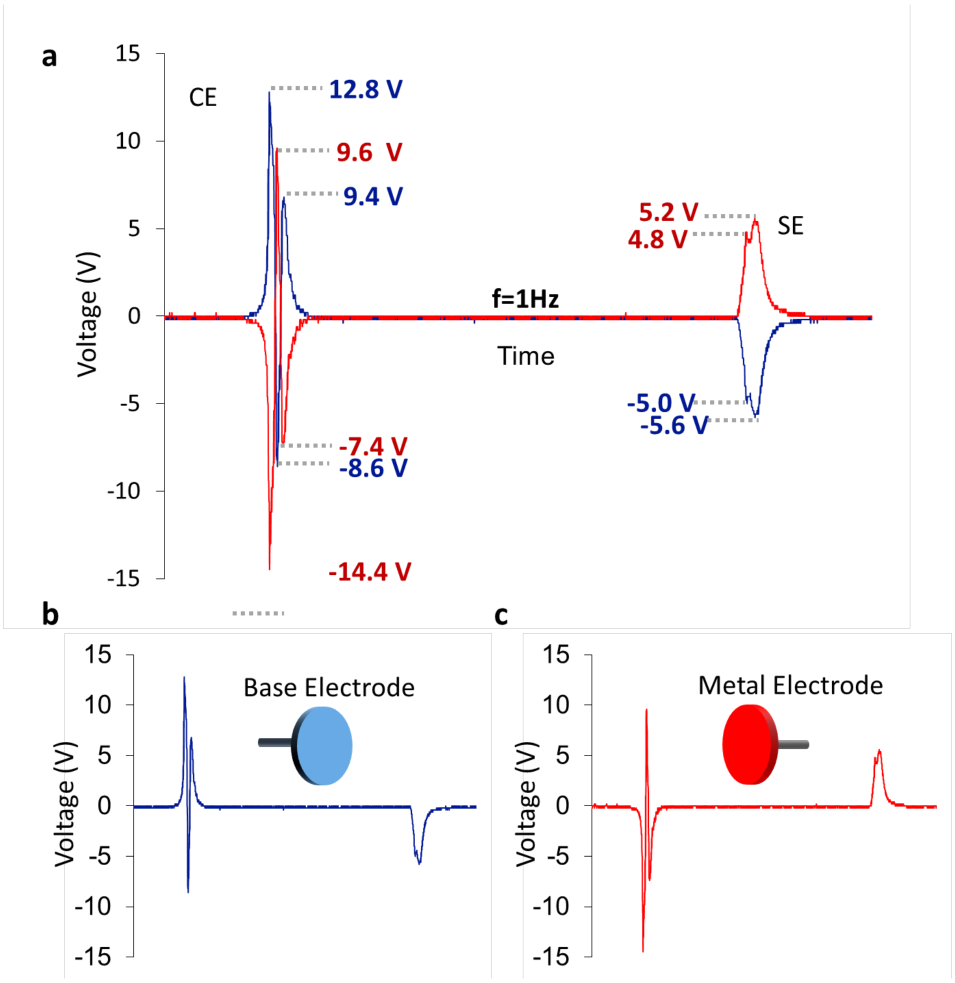

Fig S5 Open circuit potential signals produced in a contact and separation event for PDMS and aluminum metal, showing CE, SE, approach and departure inductions (I_a and I_d) on both electrodes. **a** Overall signal, **b** potential signal produced at the base electrode **c** potential signal produced at the metal electrode.

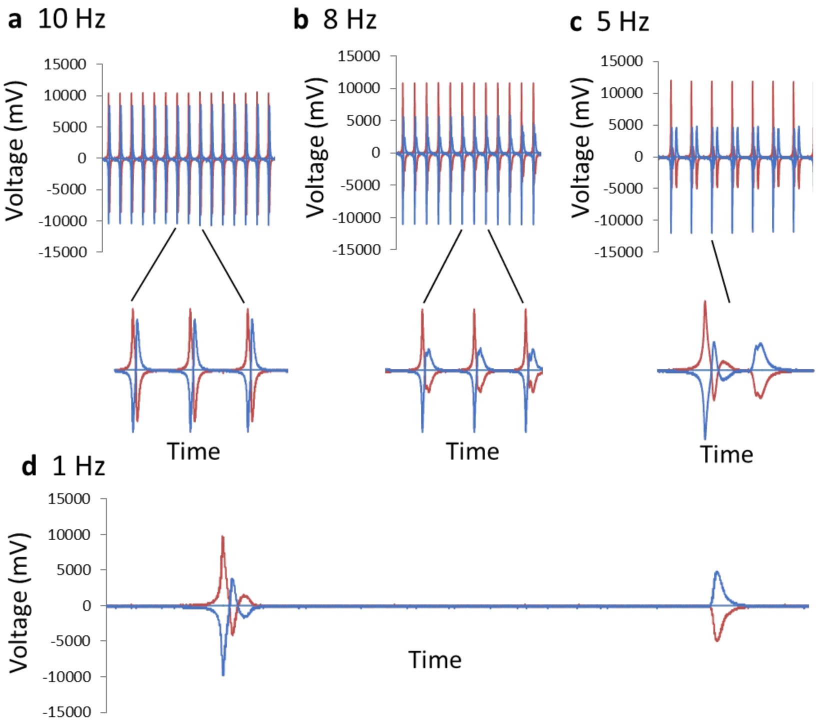

Fig S6 Open circuit potential signals produced in a contact and separation event for PVC and PET. Similar to polymer/metal electrification, the contact/separation electrification of a polymer/polymer contact gives rise to bipolar contact and unipolar separation signals at each cycle. (a-d) Generated overall potential signal for subsequent cycles at 10 Hz, 8 Hz, 5 Hz, and 1 Hz. Enlarged view of the signals for each electrode during contact and separation events showing a symmetric output w.r.t each other. In both patterns contact signals (CE signals) are bipolar (alternating + and -) and separation is unipolar.

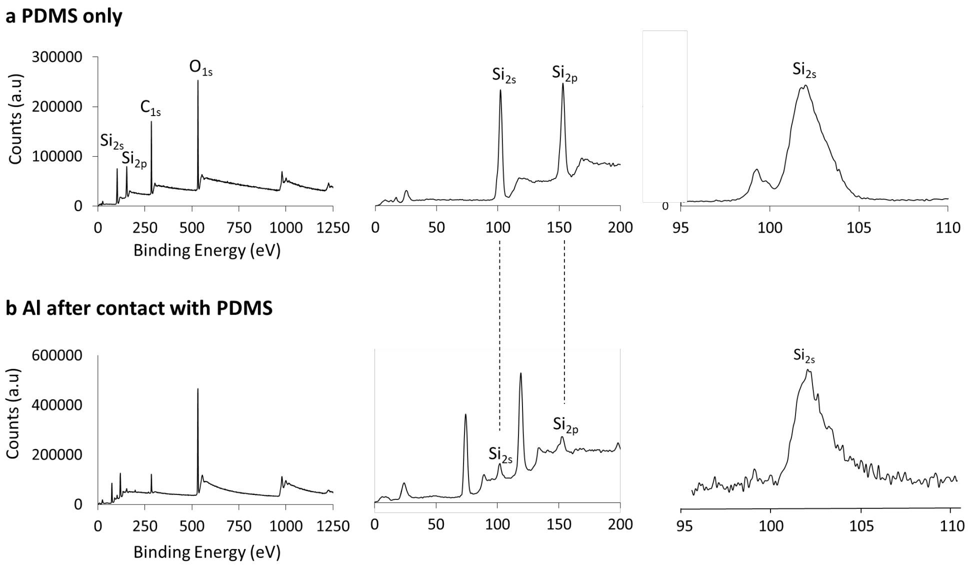

Fig S7 XPS surface analysis of Al surface after single contact and separation showing the bond breakages and transfer of PDMS on its surface. **a** the XPS signals of PDMS before contact, **b** when Al

surface after a single contact with PDMS is analyzed with XPS, Si peaks due to the material transfer from the PDMS can be observed, indicating significant amount of material transfer during contact as a result of bond breakages on PDMS. Similar examples of such material transfer and its direct relation to contact charging can be seen at ref 11.

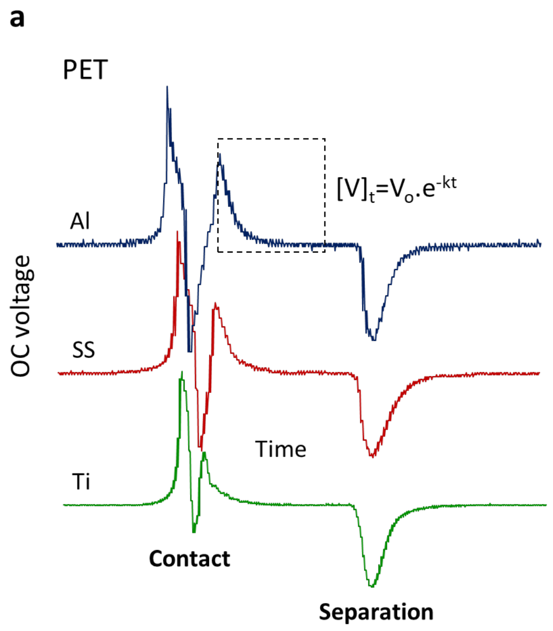

b

Base Electrode		$[V]_t=V_o.e^{-kt}$	
Metal	Polymer	V_0 (x 10^{-3})	k
**Al** $\rho=2.65\times10^{-8}$	PTFE	6.45	0.069
	PSU	14.52	0.043
	PVC	4.52	0.057
	PET	10.68	0.064
	PA	13.71	0.063
**SS** $\rho=6.90\times10^{-7}$	PTFE	9.78	0.083
	PSU	8.15	0.050
	PVC	10.03	0.091
	PET	8.49	0.043
	PA	11.62	0.079
**Ti** $\rho=4.20\times10^{-7}$	PTFE	4.60	0.048
	PSU	20.18	0.048
	PVC	10.97	0.060
	PET	17.52	0.035
	PA	3.14	0.038

Fig S8 a Various metal-polymer combinations at the BE electrode tapped at 5Hz to Al (ME). A universal behavior in signals, i.e. induction, bipolar contact charging, and unipolar separation charging signals, can be observed for all possible combinations, shown here for 'PET on Al' as BE and stainless steel (SS) as ME. **b** Discharge rates of the open-circuit potential resulting from the 'contact charges' during 5 Hz tapping of various metal-polymer combinations at the BE electrode with Al (ME) electrode, calculated from the discharge region (enlarged in dotted square) during contact charging. Data show only slight differences in discharge kinetics for different metals on BE, presumably due to the dissimilar resistivities of the metals (Al: 2.65 x10^{-8} Ωm, Ti: 4.20 x10^{-8} Ωm, and SS: 6.90 x10^{-7} Ωm). 1st order charge decay equations were generated by using curve fitting tool of MATLAB 2017b.

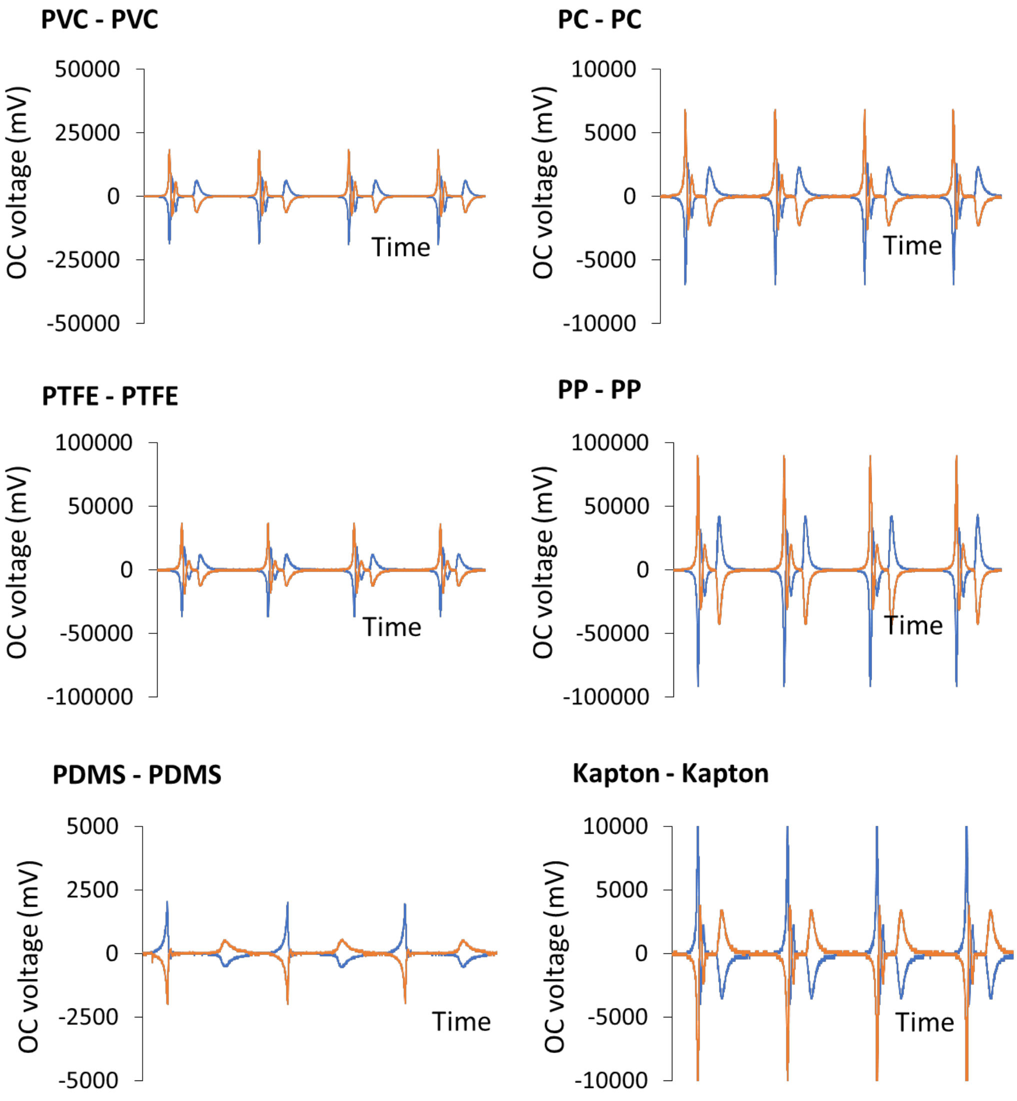

Fig S9 'Same-material, identical BE's tapping each other show that the bipolar charging and unipolar separation is not a result of charge imbalances at electrodes. Tapping frequency 5Hz; polymers mounted on Al electrode on both BE's; PVC-PVC, PC-PC, PTFE-PTFE, PP-PP, PDMS-PDMS, and kapton-kapton contacts are shown. (Tapping frequency = 5 Hz).

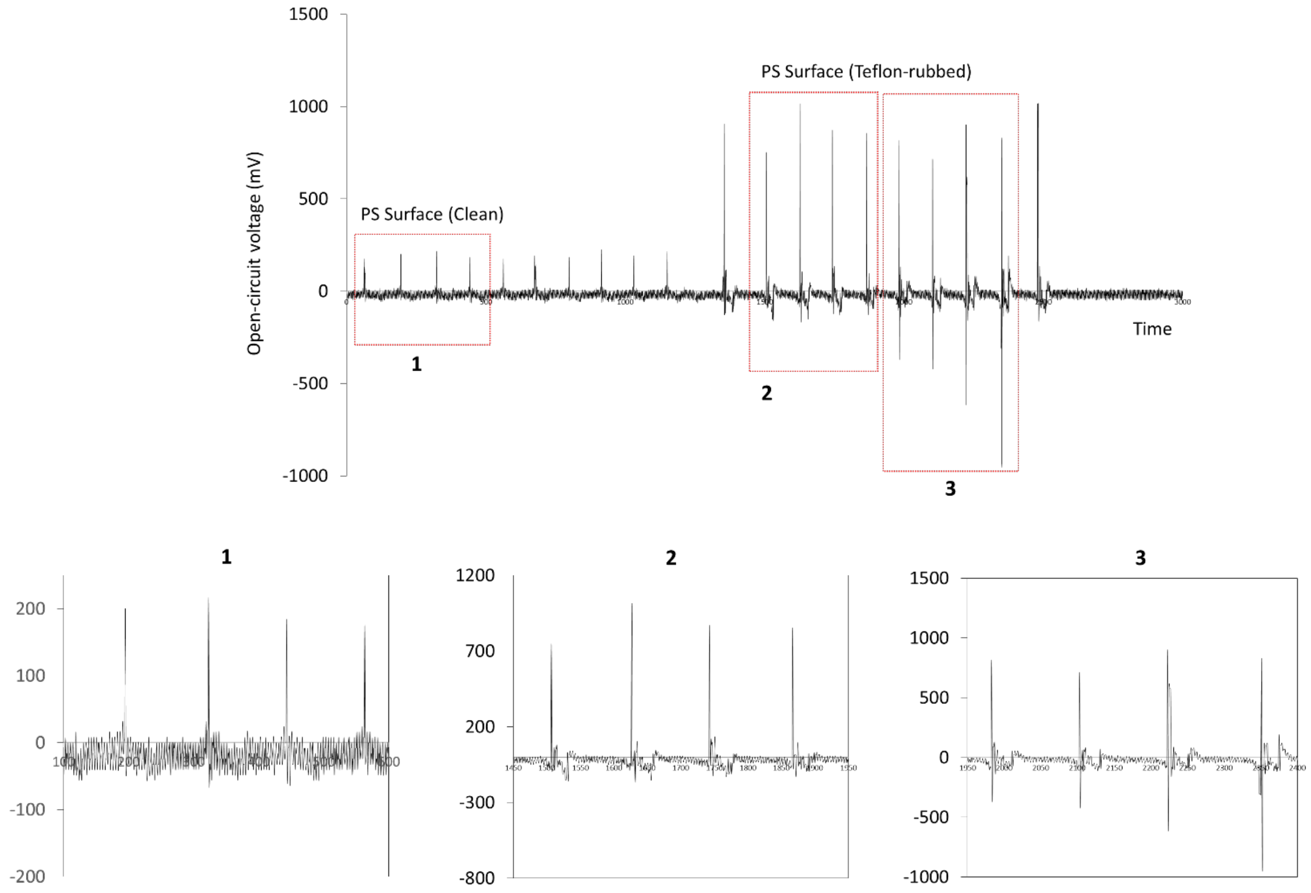

Fig S10 Spatial sampling of open-circuit voltage on a PS surface rubbed with PTFE. Rubbed PTFE has both positive and negative charge domains giving rise to a heterogeneous distribution of signals – for some signals, even to a reversal of signal polarities, while in clean PS surface the voltage distributions are more homogeneous.

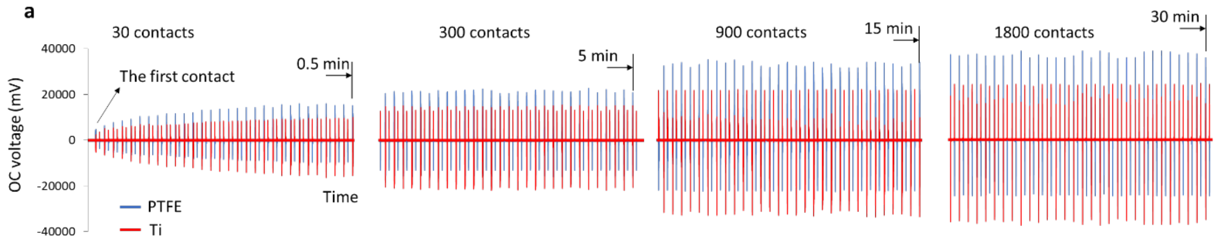

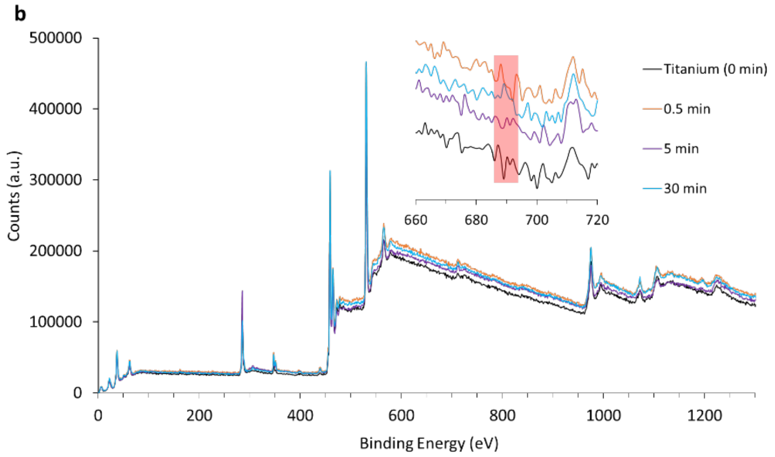

Fig S11 Effect of material transfer on signal form – PTFE-Ti contact **a** Open-circuit potential signals of consecutive contact/separation cycles upon PTFE-Ti contact recorded at 0.5 min, 5 min, 15 min, and 30 min. (PTFE on Al as BE- titanium as ME, at 1 Hz). There is no significant change in the signal forms (induction, followed by 'bipolar charging', and 'unipolar separation') with increasing tapping time. **b** There is also no significant amount of material transfer as evident from the lack of F 1s XPS signals at 690 eV (that could emerge from the transfer of bits of PTFE on Ti) on Titanium stab used as ME, even after 30 min of tapping, Fig S11b. A gradual increase in the amplitude of the open-circuit voltage signals with continued tapping (**a**), which seemed to saturate after ca. 900 contacts.

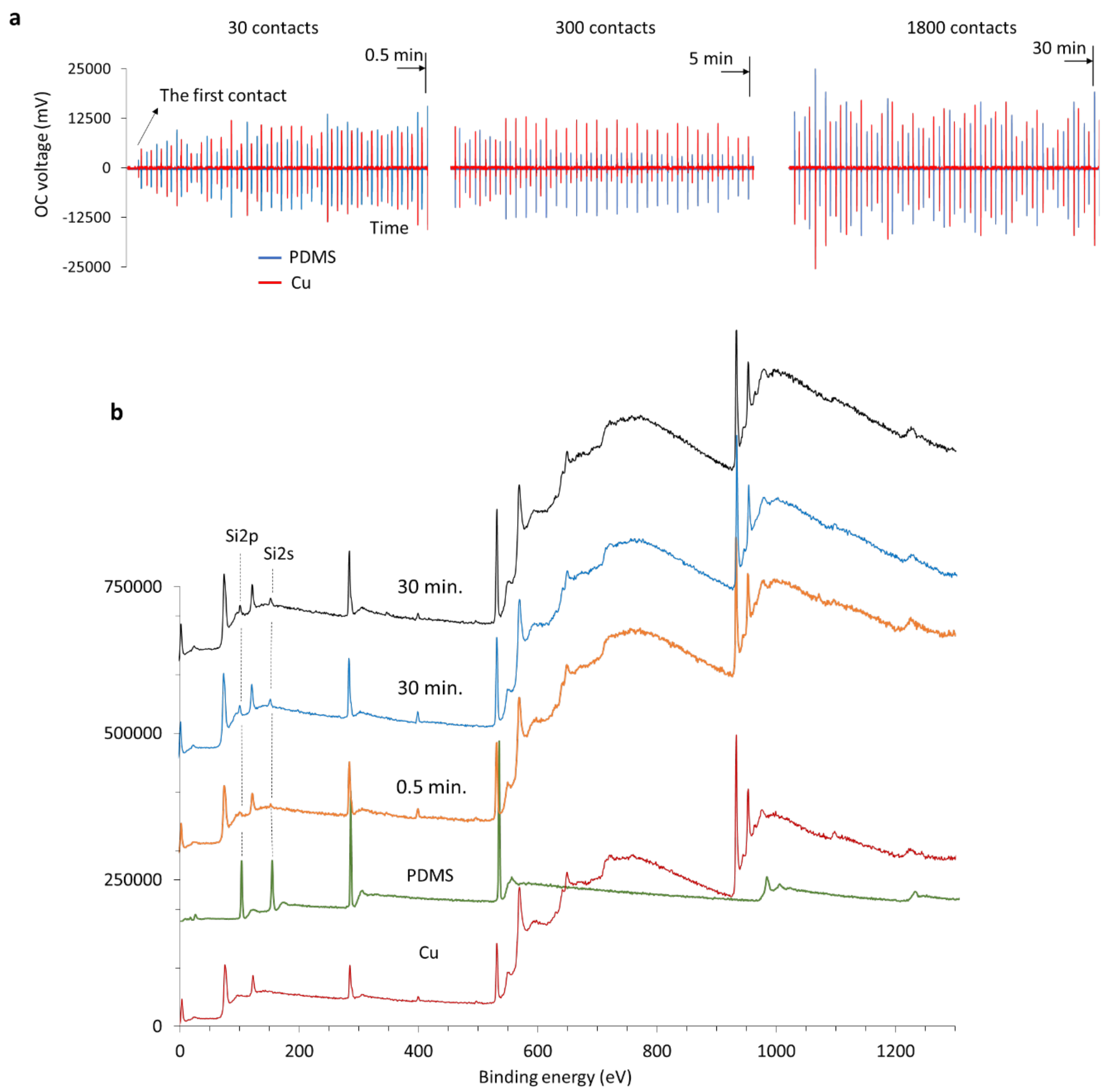

Fig S12 Effect of material transfer on signal form – PDMS-Cu contact **a** Open-circuit potential signals of consecutive contact/separation cycles upon Cu-PDMS contact recorded at 0.5 min, 5 min, 15 min, and 30 min. (PDMS on Al as BE- copper as ME, at 1 Hz). Although there is no significant change in the signal forms (induction, followed by 'bipolar charging', and 'unipolar separation') with increasing tapping time, there is a large fluctuation in the amplitude of the open-circuit voltage signals. These fluctuations seem to be a result of transferred PDMS onto Cu surface (which also allows some parts of the Cu surface to be covered with PDMS, result in heterogeneity in contact), as evident from **b** Si 2s (102.1 eV) and Si 2p (153.1 eV) XPS signals on Cu that emerged from transferred PDMS on Cu. Overall, some polymers like PDMS in this example, leave some bits of material upon contact easier than the others, the amount of which could be estimated qualitatively: Yang. L., Shirahata, N., Saini G., Zhang F., Pei L., Asplund M. C., Kurth D. G., Ariga K., Sautter K., Nakanishi T., Smentkowski V., Linford M. R. Effect of Surface free energy on PDMS transfer in microcontact printing and its application to ToF-SIMS to probe surface energies. *Langmuir* 25(10), 5674–5683 (2009), taking account the surface energy of the metal and the polymer.

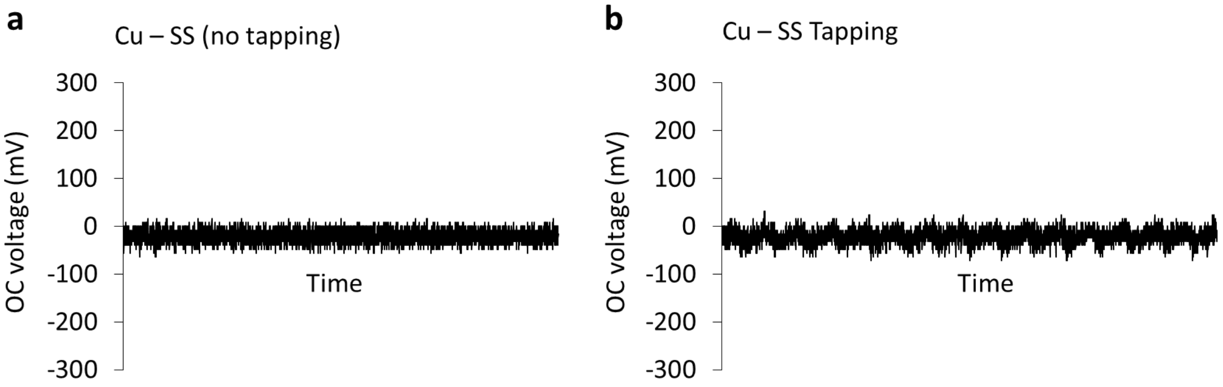

Fig S13 Open-circuit potential signals of consecutive contact/separation cycles upon Cu-Stainless steel contact.

SUPPORTING MOVIES

Movie S1. Simultaneous visual observations of corresponding signals generated at the events of contact and separation. Tapping at 1 Hz allows the identification/assignment of signals for individual events of contact and separation.

Movie S2. A grounded magnet perfectly clings (no mechanical vibrations) on the polymer piece upon release from a magnetic stop. When the magnet (***ME***) contacts the polymer surface (stainless steel on a second magnet as ***BE***) the bipolar electrical signals of *contact* are generated, similar to *contact* signals obtained from all other cases of contact/separation electrification; for polymer-metal (Fig S1, S2), or for polymer-polymer (Fig S6) contacts.

Movie S3. The effect of induction in LED circuit operation. The pins of a green light emitting diode (LED) is attached to two brass pieces each 2.5 cm x 2.5 cm surface area, which act as electrodes in a contact/separation electrification experiment. While one electrode is tapped with the polymer samples (PTFE or Nylon) the other is grounded. Note that the LED lights only during *approach-contact* OR *separation-departure* for both polymer/metal contacts - depending on the connectivity of the LED pins - since the diode allows the current only in one direction. We notice that the first contact/separation cycle of each event - which is free of electrostatic induction, I_a (as shown in Fig 3 in the main text) - does not produce any light, although there is significant electrical signal generated by the contact event. However, all the consecutive events (that produce the I_a signals) can light up the LED during *approach-contact*, showing that the LED operation is dominated by the I_a (during *approach-contact*), and the SE+I_d signals (during *separation-departure*) signals.

Movie S4 Spatial sampling of open-circuit voltage on a PS surface rubbed with PTFE. Open-circuit potential was probed by a pointy electrode touched to several different points on clean, untreated PS surface and later to some more points on the PTFE rubbed surface. The heterogeneity in the signal distributions, accompanied by reversal of potential signal signs for some points was detected on the oscilloscope signal.
